# Hypericin-Based Photodynamic Therapy Displays Higher Selectivity and Phototoxicity towards Melanoma and Squamous Cell Cancer Compared to Normal Keratinocytes In Vitro

**DOI:** 10.3390/ijms242316897

**Published:** 2023-11-29

**Authors:** Marta Woźniak, Martyna Nowak-Perlak

**Affiliations:** Department of Clinical and Experimental Pathology, Division of General and Experimental Pathology, Wroclaw Medical University, 50-368 Wroclaw, Poland; martyna.nowak-perlak@student.umw.edu.pl

**Keywords:** hypericin, photodynamic therapy, natural photosensitizer, skin cancer, melanoma, squamous cell cancer

## Abstract

The aim of this study was to explore the potential of hypericin, a naturally occurring photosensi-tizer, for photodynamic therapy (PDT) in skin cancer, investigating its phototoxic effects and mechanisms of action in cancer cells compared to normal skin keratinocytes, squamous cell cancer (SCC-25) cells and melanoma (MUG-Mel2) cells. Hypericin was applied at concentrations ranging from 0.1–40 μM to HaCaT, SCC-25, and MUG-Mel2 cells. After 24 h of incubation, the cells were exposed to orange light at 3.6 J/cm^2^ or 7.2 J/cm^2^. Phototoxicity was assessed using MTT and SRB tests. Cellular uptake was measured by flow cytometry. Apoptosis-positive cells were estimated through TUNEL for apoptotic bodies’ visualization. Hypericin exhibited a higher phototoxic reaction in cancer cells compared to normal keratinocytes after irradiation. Cancer cells demonstrated increased and selective uptake of hypericin. Apoptosis was observed in SCC-25 and MUG-Mel2 cells following PDT. Our findings suggest that hypericin-based PDT is a promising and less invasive approach for treating skin cancer. The higher phototoxic reaction, selective uptake by cancer cells, and observed proapoptotic properties support the promising role of hypericin-based PDT in skin cancer treatment.

## 1. Introduction

Hypericin is a natural compound extracted from *Hypericum perforatum* [1,2]. It exhibits various pharmacological effects, such as anti-depressive, anti-tumor, anti-inflammatory, and antiviral activity [3,4]. Several studies have demonstrated its high tumor-specific cytotoxicity, coupled with minimal side effects. Another benefit of this natural compound in tumor treatment is its ability to stimulate the generation of reactive oxygen species (ROS). Hypericin can also activate the caspase-dependent pathway, which leads to the death of cancer cells [2]. Hypericin mainly accumulates in the membranes of the endoplasmic reticulum, lysosomes, Golgi apparatus, and mitochondria due to its hydrophobic character. It has been recognized as one of the most effective photodynamic therapy (PDT) molecules [5,6].

Photodynamic therapy is a technique that uses a photosensitizer and light of a specific wavelength to specifically eliminate cancer cells in the body. The success of this therapy is determined by a variety of parameters, including the type of cancer cells, the photosensitizer (PS) applied, and the wavelength of light used for the treatment [7,8,9]. The mechanism of PDT is based on the oxidation of PS molecules under the influence of light irradiation. After PS excitation in tumor cells, the production of single ROS and free radicals begins, with cytotoxicity cascade activation, a short half-life, and a small diffusion rate, leading to apoptosis, autophagy, and necrosis of tumor cells [10,11,12,13]. PDT is considered safe, as it is less toxic than chemotherapy and minimally invasive, making it a solid, practical and promising therapeutic option for cancer treatment [14]. Numerous studies have shown that hypericin-PDT exhibits high tumor-specific cytotoxicity with few side effects, confirmed through tests such as analysis of cell viability before and after therapy and changes in cell morphology or the expression of apoptotic proteins [15,16].

Hypericin is highly light-sensitive, with maximum absorption at 590 nm, producing a significant amount of singlet oxygen and ROS (reactive oxygen species). Due to its anticancer and photochemical properties, hypericin could serve as a valuable photosensitizer for cancer treatment [5,6,17]. In conclusion, these advantages work together to make hypericin a potentially appealing photosensitizer, supported by its photochemical properties in PDT.

Skin cancer is one of the most common malignancies in the world. Also, many lesions of the skin are benign and mimic skin cancer. The most prevalent skin cancers include basal cell carcinoma, and squamous cell carcinoma [18,19,20]. Melanoma, caused by a malignancy in melanocytes, is an aggressive, relatively rare, and deadly form of skin cancer but may be curable if identified early. The number of cases of melanoma is quickly rising worldwide, causing public health problems. In 2023, over 100,000 new cases of skin cancer were detected in the United States [21,22]. Patients seek treatment for these cancers, especially due to their frequent occurrence in apparent areas, such as the face or neck [18,19,20,21,22,23]. The typical therapy for skin cancer is surgical excision [24], immunotherapy [25], gene therapy [26], chemotherapy [27], and photodynamic therapy [28].

**Figure 1:**
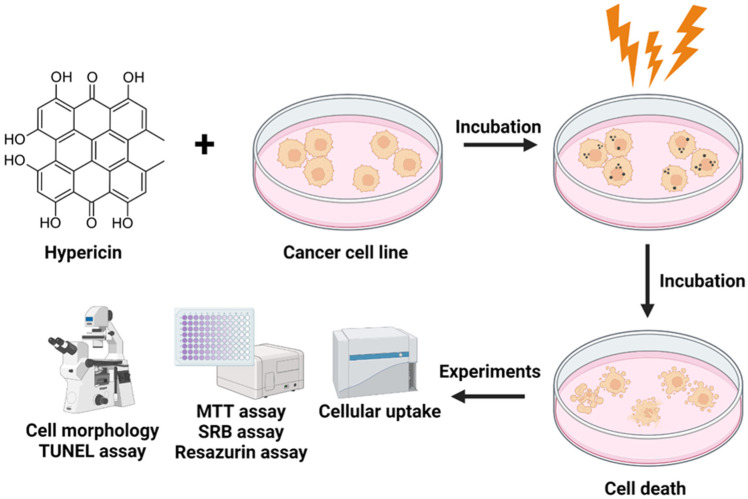
Scheme of the research methods for hypericin-mediated photodynamic therapy on skin cancer and healthy cells.

In this study (Figure 1), we investigated the phototoxic and anti-cancer effects of hypericin on human skin cancer cell lines such as SCC-25 (cutaneous squamous cell carcinoma), MUG-Mel2 (melanoma cell line), and normal human keratinocytes HaCaT as a control, including healthy cells. To evaluate the efficacy of hypericin-mediated photodynamic treatment on skin cell lines, we used MTT, SRB, and Resazurin assays to assess the natural compound’s phototoxicity. Cellular uptake was measured by using flow cytometry to demonstrate the time-dependent accumulation of hypericin in cells, and the TUNEL assay was employed to detect apoptosis.

## 2. Results

### 2.1. Cellular Uptake

**Figure 2:**
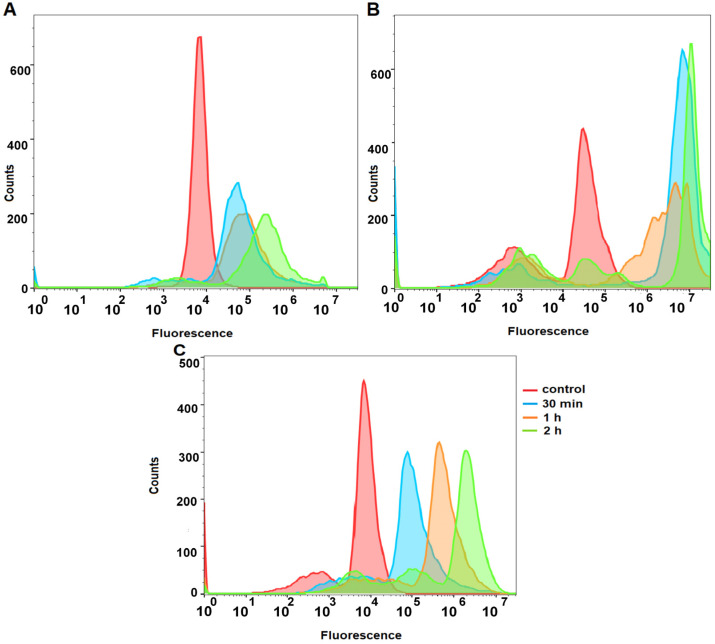
Cellular uptake was determined in HaCaT (**A**), SCC-25 (**B**), and MUG-Mel2 (**C**) cells based on the orange fluorescence of hypericin using flow cytometry. Histograms present of the fluorescence intensities of the photosensitizer in untreated cells (red line), incubated for 30 min with hypericin (blue line), incubated 1 h with hypericin (orange line), and incubated 2 h with hypericin (green line).

The time-dependent accumulation of hypericin in HaCaT, SCC-25, and MUG-Mel2 cells exposed to a 1 μM photosensitizer concentration is shown in Figure 2. The results indicate enhanced cellular uptake of hypericin in both cancer cell lines (SCC-25 and MUG-Mel2). In HaCaT cells, the uptake was lower than in cancer cells. Interestingly, MUG-Mel2 cells observed an increase in hypericin amount in cells at appropriate times. The uptake of the photosensitizer in the SCC-25 cell line presented the highest fluorescence intensity in comparison to other cell lines, but the amount of the compound accumulated in 30 min, 1 h, and 2 h was similar.

### 2.2. The Effect of Hypericin and Hypericin-Based PDT on HaCaT, SCC-25, and MUG-Mel2 Cells in the MTT Assay

**Figure 3:**
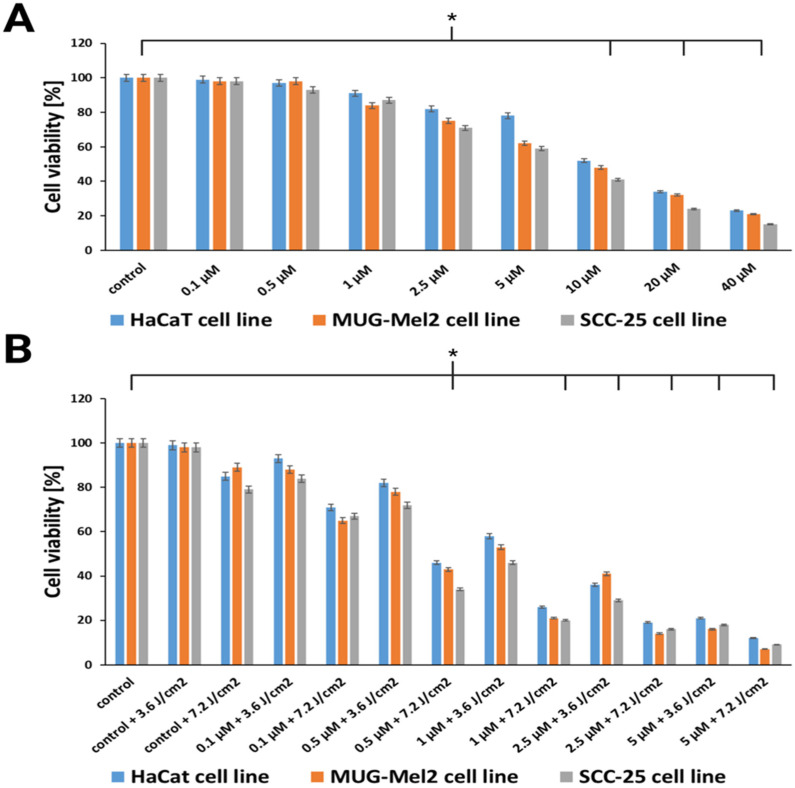
(**A**) Comparison of HaCaT, MUG-Mel2, and SCC-25 cell viability (MTT assay) after 24 h incubation with hypericin in concentrations of 0.1–40 μM. (**B**) Cell viability after treatment with hypericin-based PDT in concentrations of 0.1–5 μM after orange light in power of 3.6 J/cm^2^ or 7.2 J/cm^2^. The bars show the mean from three experiments. The statistical analysis of differences between the control and treated samples was performed using an independent samples *t*-test. * *p* < 0.05 indicated statistical significance when comparing the viability of the control cells to the MTX and Glu–MTX-treated cells. The data are expressed as the mean ± S.D of three separate experiments.

A cytotoxicity study was performed with hypericin and PDT based on hypericin focusing on normal keratinocyte cells (HaCaT), melanoma cells (MUG-Mel2), and squamous cell carcinoma (SCC-25). In the first step of our study, we conducted experiments with different doses of hypericin without irradiation (Figure 3A). Subsequently, according to our results, the experiments were conducted with the lowest doses of hypericin, which had minimal cytotoxic effects without light, and combined these small doses of hypericin with irradiation (Figure 3B). We found that hypericin and irradiation cause a higher reduction of viability in cell lines than natural substances without irradiation. PDT based on hypericin caused a higher reduction in viability in both cancer cell lines as compared to the normal cells. Therapy inhibited the growth of cancer cells in a dose-dependent manner. For example, hypericin in a 1 μM dose without light caused decreased viability in MUG-Mel2 (84% cell viability), SCC-25 (87% cell viability), and HaCaT (91% cell viability). Using the same dose of hypericin and 3.6 J/cm^2^ irradiation, cell viability decreased by 47% in MUG-Mel2, 54% in SCC-25, and 42% in the HaCaT cell line. Even higher light intensity (7.2 J/cm^2^) caused a greater decrease in cell viability in MUG-Mel2 (21% cell viability), SCC-25 (20% cell viability), and HaCaT (26% cell viability). Hypericin in the concentration of 1 μM and the irradiation power of 3.6 J/cm^2^ were chosen in all subsequent studies (cell morphology and the TUNEL assay).

### 2.3. The Effect of Hypericin and Hypericin-Based PDT on HaCaT, SCC-25, and MUG-Mel2 Cells in the SRB Assay

**Figure 4:**
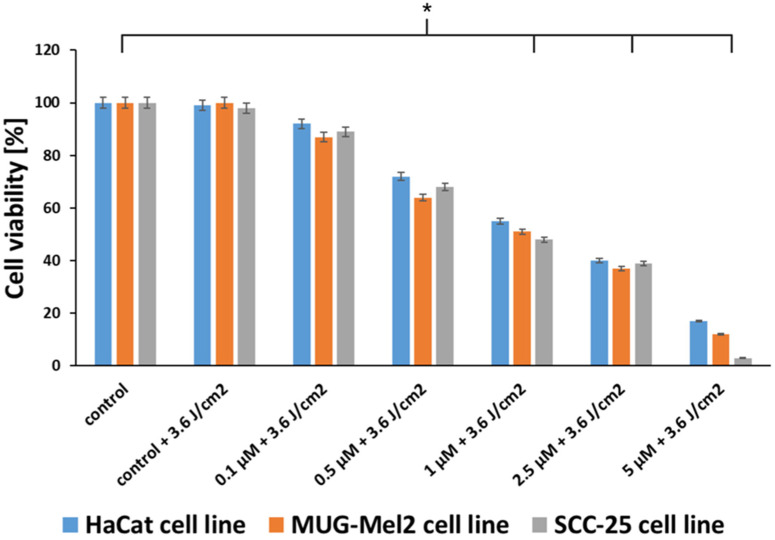
Comparison of HaCaT, MUG-Mel2, and SCC-25 cell viability measured by the SRB assay after treatment with hypericin-based PDT in concentrations of 0.1–5 μM after orange light at a power of 3.6 J/cm^2^. The bars show the mean from the three experiments. The statistical analysis of differences between the control and treated samples was performed using an independent samples *t*-test. * *p* < 0.05 indicated statistical significance when comparing the viability of the control cells to the MTX and Glu–MTX-treated cells. The data are expressed as the mean ± S.D of three separate experiments.

The SRB assay was performed with PDT based on hypericin on normal keratinocyte cells (HaCaT), melanoma cells (MUG-Mel2), and squamous cell carcinoma (SCC-25). The therapy that was used in the experiments caused a higher reduction in viability in both cancer cell lines as compared to the normal cells. Therapy inhibited the growth of cancer cells in a dose-dependent manner. Comparing the results from the MTT test, which is based on measuring the redox activity of mitochondria, to the SRB test, which consists of measuring total protein content, the effects of hypericin cytotoxicity and irradiation on cells are similar. Hypericin in the concentration of 1 μM and the irradiation power of 3.6 J/cm^2^ caused phototoxicity near IC50 (Figure 4).

### 2.4. The Effect of Hypericin and Hypericin-Based PDT on HaCaT, SCC-25, and MUG-Mel2 Cells in the Resazurin Assay

**Figure 5:**
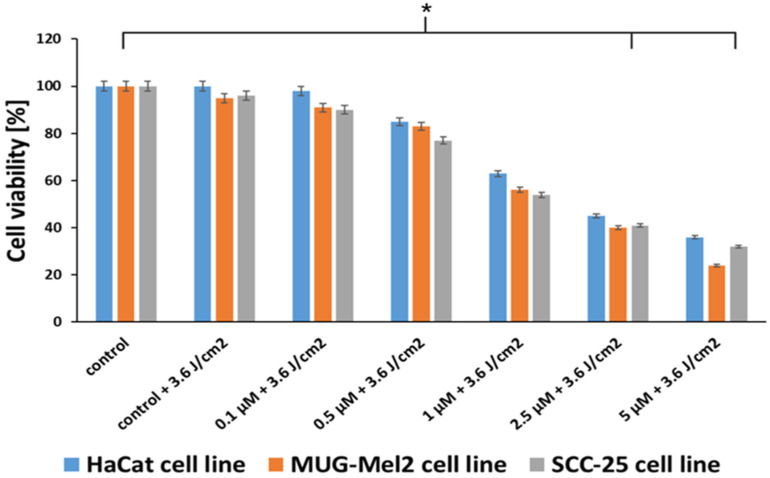
Comparison of HaCaT, MUG-Mel2, and SCC-25 cell viability (resazurin assay) after treatment with hypericin-based PDT in concentrations of 0.1–5 μM after orange light at a power of 3.6 J/cm^2^. The bars show the mean from the three experiments. The statistical analysis of differences between the control and treated samples was performed using an independent samples *t*-test. * *p* < 0.05 indicated statistical significance when comparing the viability of the control cells to the MTX and Glu–MTX-treated cells. The data are expressed as the mean ± S.D of three separate experiments.

The resazurin assay was performed with PDT based on hypericin on normal keratinocyte cells (HaCaT), melanoma cells (MUG-Mel2), and squamous cell carcinoma (SCC-25) (Figure 5). The resazurin assay allows for the discovery of living cells which are metabolically active. Thus, they are able to reduce the nonfluorescent dye resazurin to the strongly fluorescent dye resorufin via mitochondrial reductase. As shown in Figure 4, the viability of the cells (normal and cancer cells) decreased with the higher the dose of hypericin. Interestingly, comparing the results from the resazurin experiments to the MTT or SRB assays showed other outcomes. The results show that the cell viability is slightly higher than other assays. The application of a concentration of 1 μM hypericin and irradiation resulted in HaCaT cell viability of 63%, SCC-25 cell viability of 54%, and MUG-Mel2 cell viability of 56%.

### 2.5. The Effect of Hypericin and Hypericin-Based PDT on HaCaT, SCC-25, and MUG-Mel2—Cell Morphology

**Figure 6:**
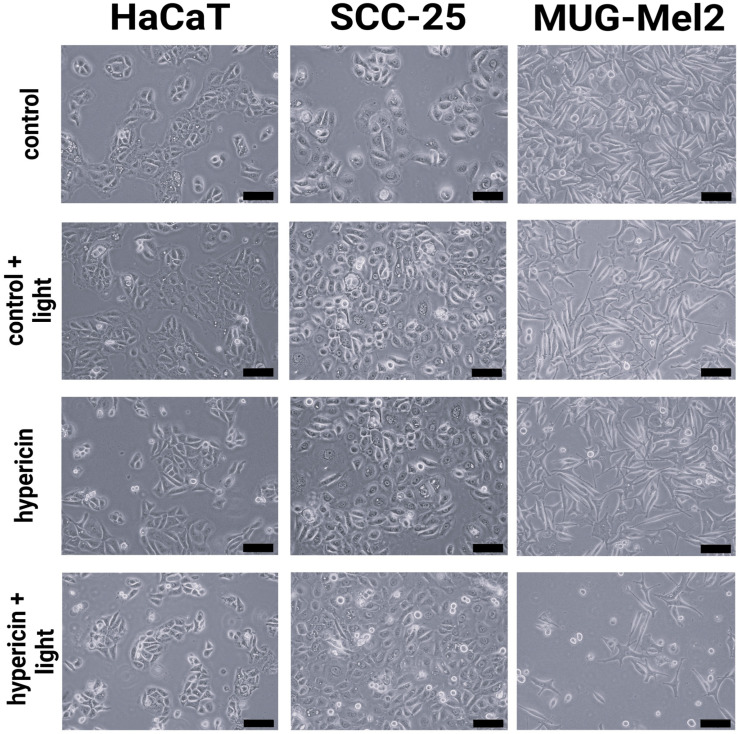
Representative images of HaCaT, SCC-25, and MUG-Mel2 cell morphology detected by phase-contrast microscopy after 24 h incubation. The image presents control cells (untreated), irradiated cells (orange light—3.6 J/cm^2^), treated cells (hypericin in dose 1 µM), and treated and irradiated cells. Scale bar = 100 µm.

Incubation of cells with high concentrations of hypericin and a portion of irradiation changed not only their functioning but also their morphology. Several images obtained using light microscopy were used to analyze the effect of the therapy used on the morphology of HaCaT, SCC-25, and MUG-Mel2 cells. The collected study indicates changes in cell activity before and after incubation. There was an increase in the number of round, detached cells (Figure 6). Throughout all experiments, similar results were observed.

### 2.6. TUNEL Assay—Hypericin and PDT Based on Hypericin on SCC-25 and MUG-Mel2 Cell Lines Induces Cell Apoptosis

**Figure 7:**
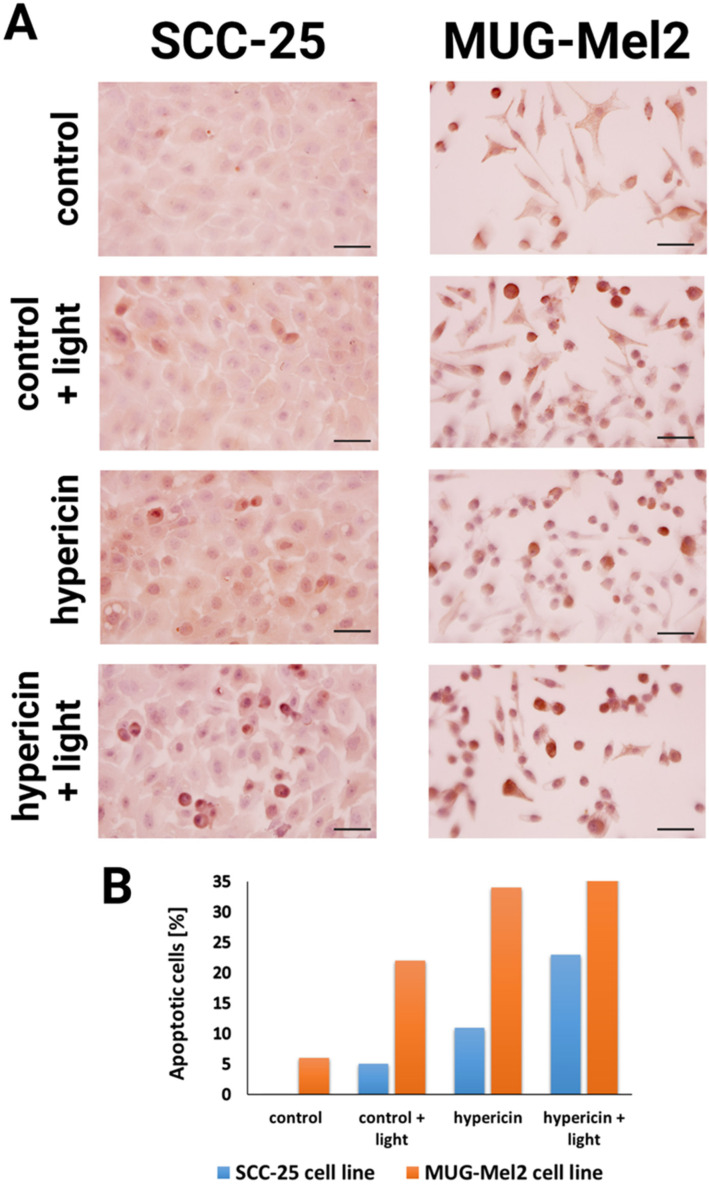
Representative photographs of the results from the TUNEL assay for the SCC-25 and MUG-Mel2 cell lines followed by photodynamic therapy (PDT) with hypericin at a dose of 1 µM with orange light (3.6 J/cm^2^) (**A**). The diagram shows the rate of apoptosis-positive cells determined by dividing the number of apoptosis-positive cells by the total number of cells in the slides × 100% (**B**). The assessment of the percentage of apoptotic cells in two cancer cell lines provides insight into the impact of light alone, hypericin alone, and the combination of hypericin and light on the apoptotic process. Scale bar = 100 µm.

We examined whether the proposed treatment of hypericin-mediated PDT leads to cell death via apoptosis. The TUNEL assay with diaminobenzidine was used to visualize apoptotic cells. Figure 7 presents an immunocytochemical analysis of the apoptotic bodies in cancer cell lines. The results show that PDT with hypericin caused the strongest apoptotic effect in MUG-Mel2 cancer cells (52% of apoptotic cells). In SCC-25 cells treated with hypericin-based PDT, 23% of apoptotic cells were detected. On the other hand, hypericin without irradiation caused a lower apoptotic effect than the combined therapy.

## 3. Discussion

Photodynamic therapy is a less invasive, alternative, and hopeful method for treating a variety of disorders, including skin cancer. Numerous PDT specialists have focused on plant extracts or natural substances as potential sources for developing efficient photosensitizers. Medicinal herbs offer an advantage over single chemicals or molecules in the treatment of many diseases due to their lower or decreased dangerous side effects [29]. The potential of hypericin as a photosensitizer in photodynamic treatment was studied in the present study on three human skin cell lines: squamous cell carcinoma SCC-25, melanoma MUG-Mel2, and immortalized keratinocyte HaCaT cells. The goals of this research were to investigate the efficiency of plant substances in photokilling skin cancer cells after low and high doses of orange light irradiation and to assess the response of healthy and cancerous skin cells (Figure 1).

According to Xu et al. and Jendželovský et al., hypericin shows beneficial phototoxic properties against adult T-cell leukemia cells and breast cancer cells, respectively [30,31]. Xu et al. [30] used a 0–200 ng/mL concentration of hypericin and a 11.28 J/cm^2^ power of light. They confirmed that the treatment with hypericin and subsequent irradiation with visible light resulted in the dose-dependent growth inhibition of all tested cell lines, whereas hypericin alone did not affect cell viability. On the other hand, Jendželovský et al. [31] used a 0–100 nM concentration of hypericin and a 3.15 J/cm^2^ power of light. Gusmão et al. and Duag et al. presented hypericin absorption spectra between 550–598 nm, with the maximum peak of absorption at 590 nm [5,6]. Therefore, for our study, we opted for orange light to irradiate cells. Moreover, Theodossiou et al. [32] and Foglietta et al. [3] confirmed that hypericin was phototoxic to the breast cancer cell lines and human colorectal adenocarcinoma used in their experiments, respectively, using light in spectra of 530–550 nm wavelengths. Their data suggested that the yellow or orange light is optimal for hypericin irradiation.

In relation to previous studies on hypericin as a photosensitizer, our results indicated this herb as a compound with lower cytotoxicity towards normal keratinocytes in comparison to skin cancer cell lines. To evaluate the most applicable dose for the experiments, the MTT method was used over a broad range of natural substance doses—from 0.1 to 40 µM for 24 h of dark incubation. For the experiments with hypericin and irradiation, we used a 0.1 to 5 µM concentration of hypericin. The IC50 of cell viability was observed at a concentration of 1 µM and low-dose irradiation (3.6 J/cm^2^) (Figure 3B). Interestingly, Popovic et al. [33] observed in normal primary human keratinocytes, melanocytes, and fibroblasts that cell viability is suppressed by hypericin-based PDT at higher doses than in the cell lines evaluated in our study. They reported an IC50 of 1.75 μM for fibroblasts, 3.5 μM for melanocytes, and 4 μM for keratinocytes, with the power of light of 5 J/cm^2^. In our results, we observed an IC50 for keratinocytes (HaCaT cells) at 1.5 μM. The difference may arise from the fact that we used primary cells isolated directly from the patients’ skin and also due to a difference in the incubation time, which was 4 h.

Another conclusion from our research is that, in normal cells (HaCaT), we observed higher cell viability than in cancer cells. Thus, the proposed therapy demonstrates lower phototoxicity against normal skin cells as compared to malignant cells. The results from the SRB assay (Figure 4) and the resazurin assay (Figure 5) confirm the data obtained in the MTT assay. Additionally, experiments were conducted to measure the ability to generate apoptosis, changes in morphology cells after the proposed therapy, and the accumulation of hypericin in three cell lines. To determine if hypericin leads to apoptosis, a TUNEL assay was performed to examine the apoptotic cells. Both cancer cell lines demonstrated that PDT with hypericin caused a stronger apoptotic effect than in untreated cells (Figure 7). Additionally, melanoma cells reveal more cells damaged by programmed cell death in comparison to squamous cell carcinoma cell lines (35% and 25%, respectively). Based on a literature review, it may be observed that hypericin followed by the irradiation of cancer cells boosted cell apoptosis in comparison to cells treated without irradiation [34,35]. To confirm apoptosis, we analyzed changes in cell morphology. As shown in Figure 6, our results revealed that hypericin-based irradiation induced apoptotic changes primarily by forming a rounder shape of cells. In the melanoma cell line, there were more apoptotic cells than in squamous cells and keratinocytes. Piryaei et al. showed that hypericin-mediated apoptosis mainly includes cytoplasmic shrinkage and forming rounder shaped cells, which corresponds to our results [35].

Cellular uptake analysis showed that hypericin was actively taken up by the studied cells. The results indicate (Figure 2) that the most significant uptake of hypericin was observed in the SCC-25 cell line. Comparable results were observed in similar experiments with emodin and aloe-emodin in our previous research [36]. The HaCaT cell line revealed the lowest uptake of hypericin, whereas MUG-Mel2 cells seemed to utilize a different strategy in which only small amounts of hypericin were taken up by the cells. Interestingly, the amount of photosensitizer in MUG-Mel2 cells after 30 min and one and two hours was at similar values. According to Damke et al. and Sattler et al., it is worth using liposomal formulations or nanoparticles to enhance increased uptake of the natural photosensitizer via cell membranes. Taken together, the exact mechanisms of the cellular uptake of hypericin are still unclear and require further investigation [37,38]. Firstly, the type of cell line affects cellular uptake. On the other hand, the literature describes different mechanisms of active and passive cellular uptake. The passive mechanism is mainly based on the temperature-dependent diffusion and solubility of photosensitizers [8]. Siboni et al. showed a direct correlation between uptake mode and intracellular distribution [39].

Taking into account that hypericin has an increased cytotoxic effect combined with PDT, recent data show the potential of natural substances in designing photoactive agents. Hypericin combined with techniques such as PDT can be a successful method of treatment for skin cancer patients. Based on our data, we believe that the cytotoxic effect of hypericin-based PDT may be a new therapeutic method in cancer treatment. However, it is worth pointing out, that the medical applications of hypericin are not easy due to several unsolved practical problems, which include poor solubility in water and sensitivity to heat and pH. Additional studies suggest creating derivatives, such as liposomes which contain hypericin to solve the solubility problem [40,41]. Further studies are required to precisely dissect the practical aspect of hypericin-based PDT in the potential treatment of skin cancer.

## 4. Materials and Methods

### 4.1. Cell Culture

HaCaT human epidermal keratinocytes (CLS, Eppelheim, Germany) were cultured in DMEM (Dulbecco’s modified Eagle medium) without calcium to maintain the normal morphogenesis and expression of the cellular membrane markers, SCC-25-tongue squamous carcinoma (DSMZ, Braunschweig, Germany) cells in DMEM-F12, and melanoma MUG-Mel2 (DSMZ, Braunschweig, Germany) cells were cultured in RPMI 1640 cell culture medium. The cell culture media were supplemented with 10% fetal bovine serum (FBS), 1% glutamine, and 1% antibiotics. Culture reagents were bought from Gibco (Thermo Fisher Scientific Inc., Waltham, MA, USA). The cells were cultured as a monolayer in 75 mL polystyrene cell culture flasks (Biologix Cell Culture Flasks, BIOLOGIX EUROPE GmbH, Niederzier, Germany). The cells were maintained at 37 °C and 5% CO_2_ in a humidified atmosphere. The cells were washed in PBS and trypsinized before being used in the experiments (0.025% trypsin and 0.02% EDTA, Sigma-Aldrich, Burlington, MA, USA). For the experiments, cells from the 3rd to the 10th passages were used.

### 4.2. Hypericin Solution Preparation

The hypericin (Biorbyt Ltd., Cambridge, UK) was dissolved in dimethyl sulfoxide (DMSO, Sigma-Aldrich) to prepare a 150 mM stock of the compound. The purity of hypericin is >98%. For the experiments, a suitable amount of stock was combined with a cell culture medium to reach the needed concentration of hypericin. The concentration of DMSO in the final solutions did not exceed 0.05%, and the highest concentration had no statistically significant effect on the cells.

### 4.3. PDT Experiment

The cells were incubated with hypericin at the required concentration for 2 h. Then, the wells were washed twice with DPBS, fresh medium was added, and irradiation was performed using a halogen lamp (Penta Lamps, Teclas, Lugano, Switzerland) with the radiation power set to 120 mW/cm^2^. Orange light was chosen to achieve the photodynamic effect (the maximum absorbance of hypericin at the wavelength of 590 nm). The cells were irradiated for 30 s (3.6 J/cm^2^). Cells exposed to hypericin and irradiation were shielded from light at all times. Before proceeding with the experiments, the cells were kept in full media during and after treatment. The experiments were carried out according to the protocol after 24 h of irradiation.

### 4.4. Cell Viability Assay—MTT Assay

The cytotoxicity of hypericin and PDT based on hypericin in HaCaT, SCC-25, and MUG-Mel2 cells was measured by the 3-(4,5-dimethylthiazol-2-yl)-2,5-diphenyltetrazolium bromide (MTT) reduction assay (Sigma-Aldrich). The cells were seeded at a density of 15 × 10^4^ per well in 96-well culture plates and treated, as described, under the experimental conditions with hypericin in doses of 0.1, 0.5, 1, 2.5, 5, 10, 20, and 40 μM for 24 h in darkness. After the treatment time, the cells were washed, and MTT solution was added to the wells to a final concentration of 1 mg/mL and the cells werw incubated at 37 °C for 2 h. The violet formazan crystals were solubilized with 50 μL DMSO (Sigma-Aldrich) for 10 min. The optical absorbance was measured at 490 nm using a BioTek ELX800 multi-well reader (BioTek, Winooski, VT, USA). The control group absorbance was 100%, whereas the treated samples’ cell viability was counted using the formula: % = (A of experimental wells/A of the control wells) × 100%.

For further MTT assays with hypericin-mediated PDT, concentrations of 0.1, 0.5, 1, 2.5, and 5 μM were used. The cells were seeded in the same way as described above. The cells were cultured with hypericin for 2 h in the dark. Following incubation, the cells were washed twice in PBS. One part of the slides was irradiated by orange light for 30 s, and another was irradiated for 1 min. The control slides were kept in the dark. Following irradiation, the cells were maintained in the complete medium for 24 h. The MTT test was performed as described above.

### 4.5. Cell Viability Assay—SRB Assay

The cytotoxicity of PDT based on hypericin in HaCaT, SCC-25, and MUG-Mel2 cells was measured by the sulforhodamine B (SRB) assay used for cell density determination, based on the measurement of cellular protein content. The cells were seeded at a density of 15 × 10^4^ per well in 96-well culture plates and treated with hypericin in doses of 0.1, 0.5, 1, 2.5, and 5 μM for 2 h in darkness. After incubation, the cells were washed twice in PBS. One part of the slides was irradiated with orange light for 30 s, and then, the cells were maintained in the complete medium for 24 h. After the incubation periods, the post-culture medium was removed, and the cells were washed with PBS. Subsequently, 50% trichloroacetic acid (TCA) was added to each well directly, and the plates were maintained at 4 °C for 1 h. The cells were washed 4 times with distilled water and dried. Next, a solution of 0.04% SRB (Sigma-Aldrich, USA) in 1% acetic acid (Avantor Performance Materials Poland, Gliwice, Poland) was added to each well. The plates were left at room temperature for 30 min. After the incubation time, the dye was removed from each well, and then, we rinsed the plates four times with 1% acetic acid to remove the unbound dye and to allow the plate to air-dry at room temperature. The SRB, which remained after washing, was solubilized in 10 mM Tris base solution (pH 10.5) on an orbital shaker for 10 min. The absorbance was measured at 510 nm using a BioTek ELX800 multi-well reader (BioTek, Winooski, VT, USA).

### 4.6. Metabolic Activity—Resazurin Assay (Alamar Blue Assay)

The cytotoxicity of PDT based on hypericin in HaCaT, SCC-25, and MUG-Mel2 cells was measured by the resazurin assay (Resazurin Cell Viability Assay Kit, Biotium, Fremont, CA, USA). The experiment was based on reducing, via mitochondrial reductase, the nonfluorescent dye resazurin to the strongly fluorescent dye resorufin by living and metabolically active cells. The cells were seeded and treated in the same way as in the SRB assay. After the experiment, to each well,10 μL of resazurin solution was added to the medium and mixed thoroughly. The plate was incubated for 2 h and 37 °C. The absorbance was measured at 570 nm using a BioTek ELX800 multi-well reader (BioTek, Winooski, VT, USA).

### 4.7. Flow Cytometry—Cellular Uptake

The cells were seeded on a 12-plate microplate (5 × 10^5^ per well). After 24 h, the cells were incubated with hypericin at a dose of 1 μM for 30 min, 1 h, and 2 h. After incubation, the cells were washed three times with warmed PBS. To each well, trypsin (250 μL) was added to detach the cells from the plate. Subsequently, a warm medium (750 μL) was added to the suspension cells and centrifuged (5 min, RT, 10,000 rpm). The supernatant was discarded and 200 µL of PBS was added to the cell pellet. Cellular uptake of the investigated photosensitizer was determined using flow cytometry (Cytoflex, Beckman Coulter Life Sciences, Indianapolis, IN, USA) and quantified based on hypericin orange fluorescence. The obtained data were analyzed using FlowJo version 10 software (BD, Ashland, OR, USA).

### 4.8. Cell Morphology

The cells were seeded in at amount of 1 × 10^6^ per well in 6-well culture plates. After 24 h, hypericin at a dose of 1 μM was added to the wells and incubated for 2 h. Following incubation, the cells were washed twice in PBS. One part of the slides was irradiated with orange light for 30 s, and then, the cells were maintained in the complete medium for 24 h. Photos were taken using an Olympus IX73 microscope with CellSens Programme (Olympus, Hamburg, Germany ).

### 4.9. TUNEL Assay—Apoptosis Assay

Apoptotic body detection after hypericin incubation with/without irradiation was evaluated by immunocytochemistry with the TUNEL assay. For the experiments, the SCC-25 and MUG-Mel2 cells were seeded on four-well chamber slides (Thermo Fisher Scientific Inc.). The cells were plated at a density of 4 × 10^4^ cells per well. The next day, hypericin at a dose of 1 μM was added to the chambers and incubated for 2 h. Following incubation, the cells were washed twice in PBS. One part of the slides was irradiated with orange light for 30 s, and then, the cells were maintained in the complete medium for 24 h. Following washing, the cells were fixed in 4% paraformaldehyde in PBS at 7.4 pH for 10 min at room temperature, and then, we kept the slide in PBS for 24 h at 4 °C. After fixation, the cells were tested following the manufacturer’s instructions using the ApopTag Peroxidase in Situ Apoptosis Detection Kit (Merck Millipore, Darmstadt, Germany). Next, the cells were washed twice with PBS, and treated with TdT enzyme for 1 h at 37 °C. The slides were washed three times in PBS and then incubated with an anti-digoxigenin peroxidase conjugate for 30 min at room temperature (RT) in a humidified environment followed by three rinses with PBS at RT. After treating the slides with the peroxidase substrate, they were counterstained with hematoxylin, dried, and mounted in a medium. The photos were captured and analyzed using a light microscope with a magnification objective of 20× (Olympus BX43 equipped with a camera and CellSens Programme, Hamburg, Germany).

### 4.10. Statistical Analysis

The results were presented as the means from the experiments performed in triplicate ± standard deviation (SD), and the statistical analysis of differences between the control and treated sample was performed using an independent samples *t*-test in the PQStat Software 1.8.6 full version program (PQStat Software 1.8.6, Poland) The differences between groups were considered significant at *p* < 0.05.

## 5. Conclusions

In conclusion, this article provides evidence that a naturally occurring compound extracted from Hypericum perforatum—hypericin—has great potential for use in photodynamic therapy as a photosensitizer for skin malignancies. Medicinal herbs are advantageous compared to chemical photosensitizers in treating various diseases because they present minimal or less severe side effects. In the presented study, we focused on three different human skin cells: squamous cell carcinoma SCC-25, melanoma MUG-Mel2, and immortalized normal keratinocytes HaCaT cells and treated them with low and high doses of orange light irradiation after exposition to different doses of hypericin. While normal skin cells show less uptake and phototoxicity after hypericin-mediated PDT, it appears to effectively induce apoptosis specifically in both skin cancer cells. However effective, it is necessary to solve issues such as low solubility, sensitivity to heat, and pH while working with hypericin by using derivatives like liposomes. Our research can also be extended to primary cell lines and explored on alternative experimental models such as spheroids or co-cultures. Taken together, the research confirms the potential of hypericin as a photosensitizer in the photodynamic treatment of skin cancer cells.

## Data Availability

All data are provided within the article.
